# Von Kossa and his staining technique

**DOI:** 10.1007/s00418-021-02051-3

**Published:** 2021-11-20

**Authors:** Marlon R. Schneider

**Affiliations:** Institute of Veterinary Physiology, University of Leipzig, An den Tierkliniken 7, 04103 Leipzig, Germany

**Keywords:** Bone, Staining methods, Histomorphometry, Von Kossa, Biography

## Abstract

One hundred and twenty years ago, the Hungarian physician Julius von Kossa developed a now classical staining method for detecting mineral deposits in animal tissues. Since then, this method has been widely adapted and combined with different counterstains, but still bears the name of its original inventor, who, if alive, would have turned 150 in 2015. As a rather inexpensive technique that does not require special instrumentation, von Kossa’s staining method became extremely popular for demonstrating mineralized tissues in vivo and in vitro. This article pays tribute to von Kossa and to his handy staining method.

## Introduction

The year 2015 marked, rather silently, the 150th anniversary of the birth of Gyula Magyary-Kossa (Julius von Kossa), a multi-talented Hungarian pharmacologist and toxicologist best known for his eponymous technique for detecting mineral deposits in animal tissues. This procedure, which remains widely used in modern histology laboratories, was published in 1901, exactly 120 years ago. This commentary briefly summarizes von Kossa’s life and assesses the original description and further developments of his staining technique.

## Julius von Kossa (1865–1944)

**Fig. 1:**
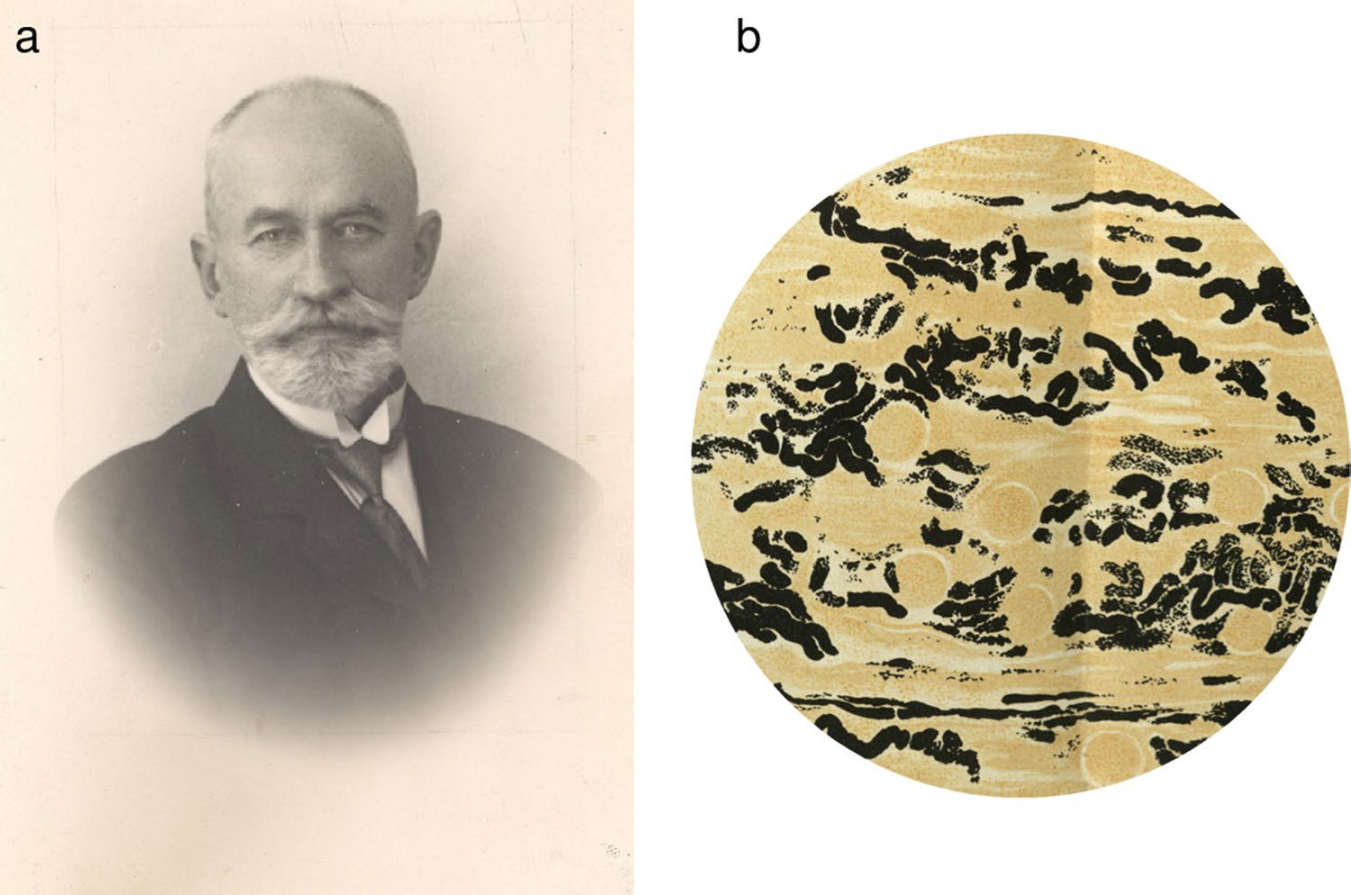
**a** Julius von Kossa (1865–1944). The photograph is courtesy of the Veterinary Science Library, Archives and Museum, Szent Istvan University, Budapest, Hungary. **b** Demonstration of lime cylinders in the kidney of a horse poisoned with copper sulphate by employing a silver nitrate staining. Reproduced from a copy of von Kossa’s original publication (von Kossa 1901) belonging to the Bavarian State Library Munich, Germany (Bayerische Staatsbibliothek)

Information about von Kossa’s life is scarce and, to my knowledge, limited to short articles in Hungarian, which in part present conflicting information. I briefly summarize the facts these reports agree on, thereby adhering essentially to the information presented by Dénes Karasszon in 1990 (Karasszon 1990). Von Kossa (Fig. 1a) was born on 8 January 1865 in Debrecen, Hungary. After attending schools in different Hungarian cities, he was admitted in 1883 to the Faculty of Medicine of Budapest University. After his graduation in 1889, he obtained different academic positions (most likely corresponding to modern postdoctoral positions) before becoming an associate professor of the faculty in 1894. Two years later, he founded and became the head of the Department of Pharmacology at the Hungarian Royal Veterinary Academy, a position he would occupy for 40 years. He was strongly attracted to toxicological studies, which therefore represent a major topic of his publications. For instance, he discovered effective antidotes against morphine and cyanide poisoning, and he was one of the first investigators to demonstrate that pental, then a widespread anaesthetic, could lead to fatal heart side effects. Other interests included librarian activities (he was responsible for the library of the Hungarian Royal Veterinary College from 1901 to 1909) and medical history: three large volumes focussing on Hungarian medical history appeared between 1929 and 1931. He possessed vast classical and modern language skills, and seemed to be a talented piano player. Von Kossa also received several important honours and awards, including the appointment as a *Hofrat* (royal court adviser). He retired in 1936 after 47 years of academic duties in teaching and research. He died at the age of 79, on 21 June 1944, apparently in Keszthely, a small town located on the western shore of Lake Balaton, and was buried at Kerepsi, one of the oldest cemeteries in Budapest. In 1990, a bronze bust of von Kossa was unveiled in the park of the University of Veterinary Medicine of Budapest, the major place of his activity.

Von Kossa’s life coincides with the sweeping changes taking place in the Hungarian capital at the transition from the nineteenth to twentieth century. The House of Habsburg, one of the most prominent royal houses of Europe in the second millennium, had ruled Budapest for centuries. They introduced German as the official language in the eighteenth century, thus in part explaining the publication of von Kossa’s work in this language (German was, anyway, the lingua franca of Western science from the late nineteenth century, losing its leading position at the end of World War II). Following the permanent unification of Buda and Pest in 1873, the population of Budapest grew at an amazing pace (it actually doubled between 1869 and 1896), becoming the eighth largest city in Europe by 1910 (Voros 1997). Budapest became one of the major cultural centres of Europe, comparable to Vienna or Paris before World War I extinguished the flourishing *belle époque*. After a temporary recovery between the inter-war years, Budapest was literally devastated by World War II; it seems that the residence of von Kossa at Keszthely at the time of his death was an attempt to flee the horrors of war.

## The original description of von Kossa staining

Today, von Kossa’s name is associated with a staining technique for detecting mineral deposits in different tissues. The article, dated June 1900, was published in 1901 in the 29th volume of *Beiträge zur pathologischen Anatomie und zur allgemeinen Pathologie* with the title “About artificially generable calcifications in the organism” (“*Ueber die im Organismus künstlich erzeugbaren Verkalkungen*”) (Kossa 1901). It comprises 39 printed pages (issue pages 163–202) and a plate including six colour figures. After a compilation of the then-known facts about renal calcification induced by ischemia and reperfusion or by a number of toxins, the author describes and interprets the results of a number of personal experiments on the induction of calcification by diverse toxins, mostly in rabbits and horses (pages 163–178). The next part of the article deals with chemicals and histological methods for detecting mineral deposits in tissues, with a focus on kidney and liver (pages 178–193). It is here, between pages 188 and 191, that von Kossa describes his motivations and the method he developed to stain mineral deposits:

“My ambition was to find a staining method that would, at the same time, be usable as a microscopic reaction of lime, or rather phosphate of lime, without causing the slightest change in the form of the lime cylinders or lime grains. After a demanding search, this succeeded entirely.” (“*Mein Streben war gleichzeitig, eine Methode der Färbung zu finden, welche gleichzeitig als mikrochemische Reaction des Kalkes, beziehungsweise des Kalkphosphats, verwendbar wäre, ohne dass die Gestalt der Kalkzylinder oder Kalkkörner im Geringsten geändert würde. Nach langwierigem Suchen gelang dies vollkommen*”; page 188).

In the key experiment, von Kossa treated sections of kidney with 1–5% solutions of silver nitrate, which resulted in a yellow coloration of calcium cylinders. This coloration was transient and gradually changed through grey to black. To illustrate his findings, he provides an impressive picture of the kidney of a horse poisoned with copper sulphate; the silver nitrate staining allows a clear discrimination of the calcium cylinders (Fig. 1b). In the remaining part of the article, von Kossa considers and investigates the question of whether the calcium deposited in the different tissues derives from the bones and discusses possible causes of tissue mineralization.

## Chemical basis of the staining

In the decades preceding von Kossa’s discovery, organic chemistry underwent a remarkable transition. Initially overlapping with plant and animal natural history and with a strong focus on commercial exploitation, “carbon chemistry” evolved to a “productive experimental culture”, focussing on chemical reactions and the quantitative analysis of organic compounds after ~1840 (Klein 2003). This novel (sub-)discipline, characterized by technical and semiotic (essentially the use of Berzelian formulas) innovations as well as by a mushrooming of professional chemists, chemical journals and societies, progressed astonishingly. Nineteenth-century chemists were therefore quite familiar with reactions and colour changes of silver salts in contact with organic substances. Silver nitrate, given internally to treat a number of diseases including epilepsy and some forms of syphilis, caused dark grey coloration of the skin and internal organs (argyria), and this silver salt was also introduced into histology to demonstrate cell borders in the second half of the nineteenth century (Puchtler and Meloan 1978).

Von Kossa’s technique is clearly a two-step reaction: first, silver cations react with components of calcium deposits, resulting in a transient yellow coloration, while in the second step organic material reduces the bound silver to black metallic silver with the aid of light or by photographic developers. Notably, as already highlighted by other authors (Puchtler and Meloan 1978; Meloan and Puchtler 1985), von Kossa was fully aware that only the yellow staining in early stages of the reaction is diagnostic for calcium phosphate, and he correctly regarded the posterior blackening as a secondary reaction caused by organic matter. This important distinction was lost in several later studies. It would be beyond the scope of this commentary to review the chemical basis of von Kossa’s reaction and its many adaptations in detail; the interested reader is referred to articles dealing with these aspects (Puchtler and Meloan 1978; Meloan and Puchtler 1985). In particular, Puchtler and Meloan (1978) present the relevant chemical knowledge available at von Kossa’s time and his own chemical interpretation of the findings.

## Further developments and current use

**Fig. 2:**
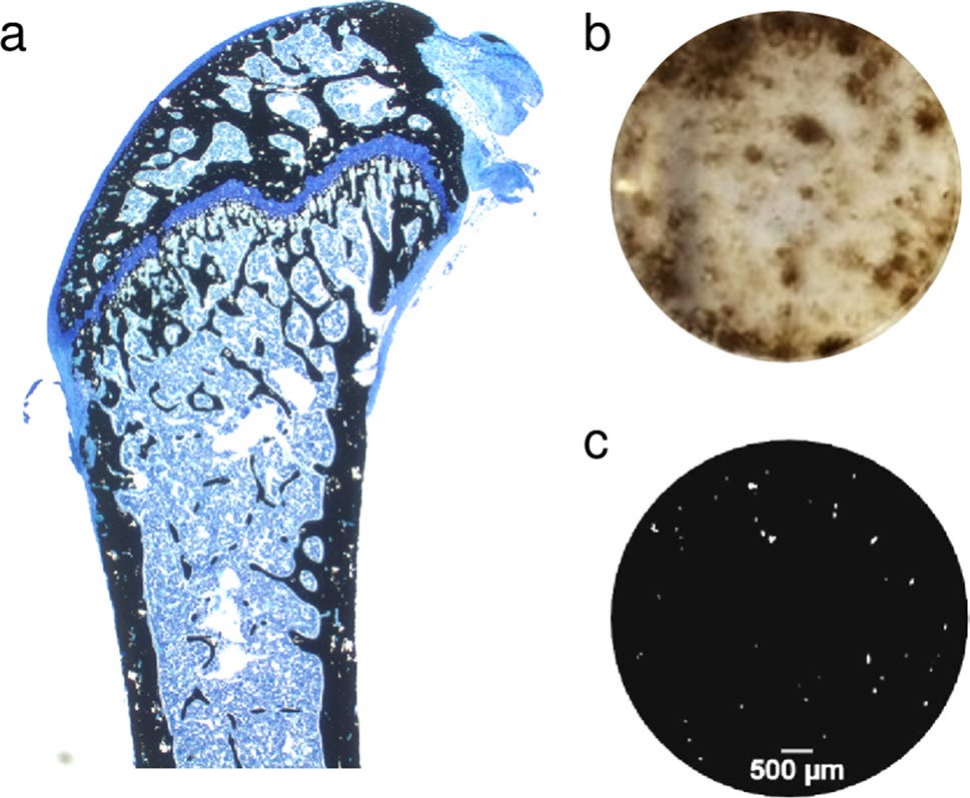
**a** Longitudinal sections of distal femur of a 9-week-old female mouse. Microtome sections (3 mm thick) from MMA-embedded bones were stained with von Kossa/McNeal’s tetrachrome. Reproduced from Schneider et al. (2009). **b** Mineralization of osteoblast cultures demonstrated by von Kossa staining. **c** Visualization of bone resorption after culture of osteoclasts on a resorbable calcium phosphate substrate using von Kossa staining. **b** and **c** were adapted from Estell et al. (2020), licensed under https://creativecommons.org/licenses/by/4.0/

Von Kossa’s technique was soon adapted and further improved and became, in combination with diverse counterstains, a standard method for staining bone and other mineralized tissues. For instance, in combination with MacNeal’s tetrachrome counterstain (Fig. 2a), it is widely used for static cancellous histomorphometry in rodents, as it provides good cellular detail and a sharp distinction between mineralized and un-mineralized bone (Ma et al. 2020). It can also be employed for assessing mineralization in cultured cells (Fig. 2b), and to (indirectly) assess bone resorption. In the latter method, bone resorption pits created by osteoclasts appear as white spots against the homogeneous black background produced by staining a resorbable calcium phosphate substrate with the von Kossa method (Fig. 2c). Its major disadvantage is that the dark precipitate obscures structural details, such as lamellae or cement lines within the calcified matrix, precluding more informative quantitative analysis of bone sections (Schenk et al. 1984). Another limitation is its poor specificity, and caution should be exercised when interpreting von Kossa staining as a measure of mineralized bone matrix formation in vitro. For instance, positive staining observed in osteoblast cultures may not represent hydroxyapatite, but instead correspond to dystrophic mineralization of unknown origin (Bonewald et al. 2003). Despite these disadvantages, von Kossa’s staining technique became the most popular method for detecting mineralized animal tissues, and immortalized its almost forgotten discoverer.
